# Single subanesthetic dose of ketamine produces delayed impact on brain [^18^F]FDG PET imaging and metabolic connectivity in rats

**DOI:** 10.3389/fnins.2023.1213941

**Published:** 2023-07-13

**Authors:** Sarah Chaib, Caroline Bouillot, Sandrine Bouvard, Benjamin Vidal, Luc Zimmer, Elise Levigoureux

**Affiliations:** ^1^Université Claude Bernard Lyon 1, Lyon Neuroscience Research Center, CNRS, INSERM, Lyon, France; ^2^Hospices Civils de Lyon, Lyon, France; ^3^CERMEP-Imaging Platform, Bron, France

**Keywords:** ketamine, FDG, PET, metabolic connectivity, neuroimaging

## Abstract

**Introduction:**

Ketamine, a glutamate NMDA receptor antagonist, is suggested to act very rapidly and durably on the depressive symptoms including treatment-resistant patients but its mechanisms of action remain unclear. There is a requirement for non-invasive biomarkers, such as imaging techniques, which hold promise in monitoring and elucidating its therapeutic impact.

**Methods:**

We explored the glucose metabolism with [^18^F]FDG positron emission tomography (PET) in ten male rats in a longitudinal study designed to compare imaging patterns immediately after acute subanaesthetic ketamine injection (i.p. 10 mg/kg) with its sustained effects, 5 days later. Changes in [^18^F]FDG uptake following ketamine administration were estimated using a voxel-based analysis with SPM12 software, and a region of interest (ROI) analysis. A metabolic connectivity analysis was also conducted to estimate the immediate and delayed effects of ketamine on the inter-individual metabolic covariance between the ROIs.

**Results:**

No significant difference was observed in brain glucose metabolism immediately following acute subanaesthetic ketamine injection. However, a significant decrease of glucose uptake appeared 5 days later, reflecting a sustained and delayed effect of ketamine in the frontal and the cingulate cortex. An increase in the raphe, caudate and cerebellum was also measured. Moreover, metabolic connectivity analyses revealed a significant decrease between the hippocampus and the thalamus at day 5 compared to the baseline.

**Discussion:**

This study showed that the differences in metabolic profiles appeared belatedly, 5 days after ketamine administration, particularly in the cortical regions. Finally, this methodology will help to characterize the effects of future molecules for the treatment of treatment resistant depression.

## Introduction

Ketamine is a dissociative anesthetic agent that has been widely used in medicine since the 1960s. Recently, this glutamate receptor antagonist has been the subject of numerous studies for the treatment of drug resistant unipolar depression at subanesthetic doses (Krystal et al., 2019). By inhibiting N-methyl-D-aspartate receptors (NMDAR), ketamine is believed to produce rapid-acting and robust antidepressant effects (Berman et al., 2000; Zarate et al., 2006).

In 2019, a nasal form of the S(+) enantiomer of ketamine, also known as esketamine, was developed and approved as a medication by the European Medicines Agency (EMA) and the Food and Drug Administration (FDA) in the USA for treatment-resistant depression (TRD) and MDD (Sanacora et al., 2017).

However, ketamine can cause a variety of side effects (such as psychiatric and cardiovascular disorders), and its status as a narcotic with abuse potential makes its use complex (Bonaventura et al., 2021). In order to assist in the development of new rapid-acting antidepressants (RAADs) (Duman, 2018), a better understanding of the mechanisms involved in the sustained action of subanesthetic doses of ketamine is needed (Witkin et al., 2019). While many studies focused on the molecular and cellular mechanisms of ketamine (Li et al., 2010; Autry et al., 2011; Niciu et al., 2014; Park et al., 2014), less studies have investigated its action at the scale of the entire brain. Moreover, specific translational biomarkers of ketamine effects are still needed both in animal models and humans.

In this regard, neuroimaging techniques are a valuable tool for investigating *in vivo* brain activity in both rodents and humans. An example of a commonly used technique for studying cerebral activity at macroscale is observing and quantifying glucose metabolism in the brain using [^18^F]-fluoro-2-deoxy-D-glucose (FDG) PET (positron emission tomography). [^18^F]FDG is a radiolabeled glucose derivative, whose brain uptake is highly correlated to brain function, that has been largely used for the explorations of psychoactive drugs and their mechanism of action (Schöll et al., 2014; Levigoureux et al., 2019) and notably ketamine (Vollenweider et al., 1997; Långsjö et al., 2004; Radford et al., 2018; Chen et al., 2022). These clinical and preclinical studies find metabolic modifications in similar regions, in particular the anterior cingular cortex (ACC), the thalamus, and the frontal cortex, supporting the translational aspect of this method. Other PET studies using specific radiotracers have been carried out on various neurotransmitter systems involved in the ketamine mechanism (Salmi et al., 2005). For instance, a clinical study using [^11^C]ABP688 showed a decrease of mGluR5 receptor availability after ketamine administration (Esterlis et al., 2018) and a preclinical PET study using [^18^F]FDOPA revealed an increase in dopamine synthesis (Halff et al., 2022).

Other complementary imaging techniques can be also used as biomarkers of the effects of ketamine. Indeed, several functional magnetic resonance imaging (fMRI) studies described regional effects of subanesthetic doses of ketamine in frontoparietal regions, ACC or hippocampus [reviewed in Ionescu et al. (2018)].

However, these studies have mainly focused on the immediate effects of ketamine. Therefore, we propose a novel design using metabolic imaging with [^18^F]FDG PET in rats to compare changes in glucose brain metabolism after acute subanaesthetic ketamine injection (10 mg/kg) with its sustained effects (5 days later). In addition to explore changes in radioactive patterns, we also investigated changes in metabolic connectivity, as a way to examine the effects of ketamine on the interaction between different brain regions. This method has been shown to provide valuable additional insights into brain metabolism [reviewed in Yakushev et al. (2017)], similarly to the functional connectivity analysis in fMRI.

## Methods

### Experimental animals

A total of ten adult male Sprague–Dawley rats (Charles River laboratories, France) weighing 225–250 g were used in the present study. Animals were received in 3 different batches and the rats were housed between two and four per cage in standard temperature (22 ± 2°C) and humidity (50%) conditions with a 12 h/12 h light/dark cycle, light on at 8.00 am; food (Teklad 18% protein rodent diet, Envigo, USA) and tap water were provided *ad libitum*. All experiments were performed in accordance with the European guidelines for care of laboratory animals (directive 2010/63/EU) and approved by the University of Lyon review board. An initial acclimatization period of 5 days was observed before scans.

### PET imaging procedures and data analyses

#### PET/CT protocol and image acquisition

**Figure 1:**
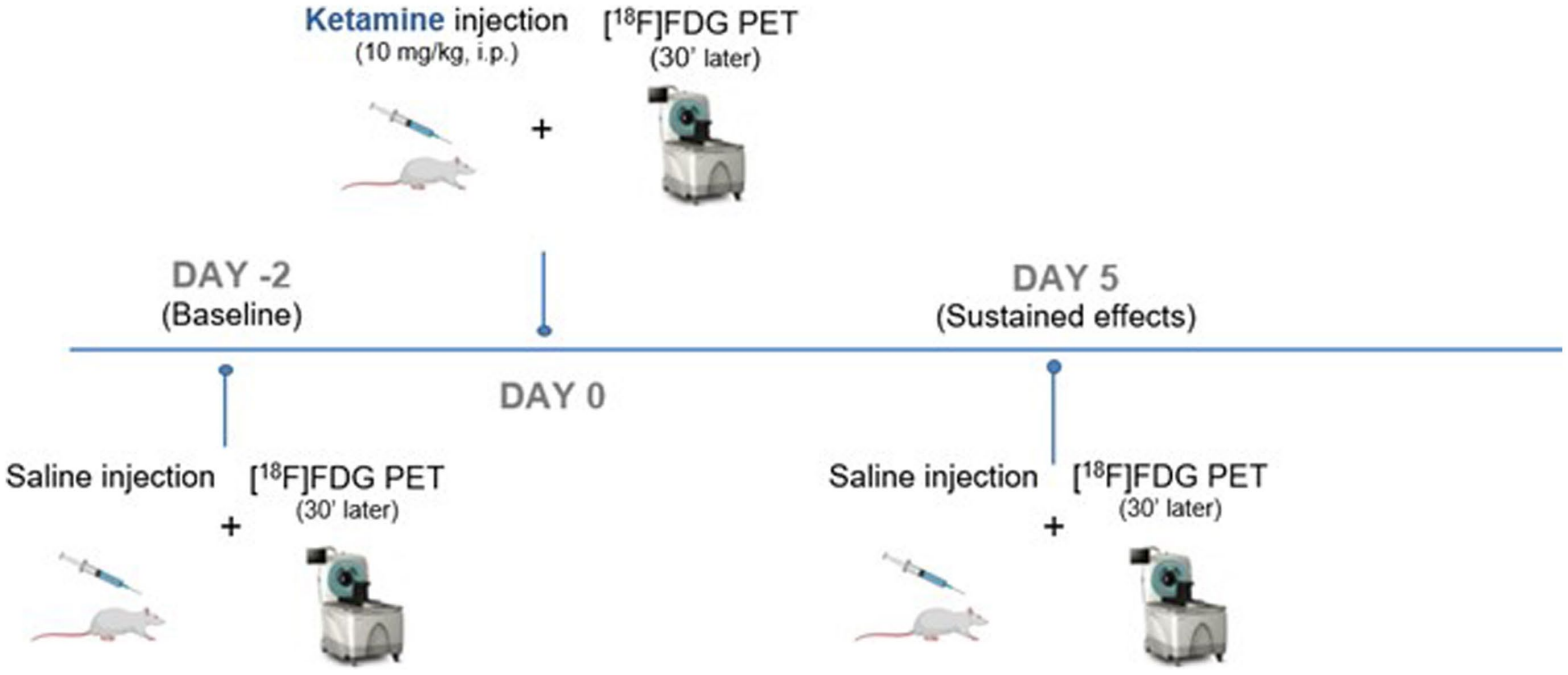
Timeline and PET study design.

A longitudinal design was used with each animal that underwent 3 different PET scans with [^18^F]FDG (Figure 1): a first one representing the baseline (Day −2), a second 48 h later (Day 0) with a subanesthetic dose of ketamine and a final one, 5 days after ketamine (Day 5). Ketamine®1,000 (100 mg/mL) was obtained from Virbac, France and dissolved in saline at a dose of 10 mg/kg, qsp 0.5 mL. Rats were fasted for 4 h before each scan. Fifteen minutes after *i.p.* injection of 0.5 mL of ketamine (at Day 0) or saline (at Day −2 and Day 5), the caudal vein was catheterized and [^18^F]FDG (38.94 ± 2.5 kBq/g) was injected under awake conditions. Thirty minutes later, rats were anesthetized with constant insufflation of isoflurane (4% for induction then 2% during acquisition, 1 L/min) and placed in the PET-CT imaging scanner (INVEON®, Siemens). PET scans were acquired in list mode during 30 min, completed by a computed tomography (CT) scan for the tissue attenuation correction. Static images of the 30 min acquisitions were reconstructed with attenuation and scatter corrections. The reconstructed volume was constituted of 159 slices of 128 × 128 voxels, in a bounding box of 49.7 × 49.7 × 126 mm^3^ and with voxel size 0.388 × 0.388 × 0.796 mm^3^ OSEM 3D.

#### PET data analyses

Data processing was carried out using INVEON Research Workplace (IRW®, Siemens) software, and the statistical parametric mapping software SPM12®. Images were analyzed in two steps, i.e., a voxel-based analysis and a regions of interest (ROIs) analysis.

##### Voxel-based analyses (VBA)

Individual PET images were realigned and spatially normalized based on our own previously built CT template, co-registered on an anatomical MRI template. Each PET volume was then spatially normalized and smoothed using an isotropic Gaussian filter [1 mm × 1 mm × 1 mm]. Local voxel activities were normalized to the mean uptake of the whole brain taken as a unique ROI. In other words, the activity in each voxel was divided by the average value of the whole brain. A voxel-to-voxel statistical analysis was performed to compare the metabolic profiles, using a variance analysis in a generalized linear model, to compare mean uptakes between the different conditions. This statistical analysis resulted in activation (Contrast: Day 0 or Day 5 – baseline] and inhibition ([Baseline – Day 0 or Day 5]) maps. A significant threshold was set up at *p* < 0.001 uncorrected, at the voxel level.

##### Regions of interest (ROIs) analyses

Eight brain ROIs were selected from the Lancelot rat brain atlas on the corresponding MRI template (Lancelot et al., 2014), as being potentially involved in the mechanism of action of ketamine. Mean uptake ratios were extracted in the different ROIs, and a two-way mixed ANOVA for repeated measures (factors: time and ROI) with Tukey’s post-hoc tests (corrected for multiple comparisons) were performed using GraphPad Prism 8.0 software to compare the [^18^F]FDG uptake ratios between the different conditions.

To minimize the variability and account for potential confounding factors such as changes in peripheral glucose metabolism, the standardized uptake values in each scan were normalized to the mean radioactivity in the whole brain, in order to ensure a consistent comparison of regional effects. Because this approach may underestimate global changes in brain metabolism, we also reported the mean standard uptake values (SUVs).

#### Metabolic connectivity analysis

For each day (Baseline, Day 0, Day 5), pairwise regional correlations across subjects were evaluated by the Pearson correlation coefficient. For statistical comparisons of correlation matrices between the different days, we used a permutation testing method as previously described (Choi et al., 2015) using Matlab (R2023a, Mathworks). Briefly, correlation coefficients were transformed into Fisher Z scores. For each comparison (Baseline vs. Day 0, Baseline vs. Day 5, Day 0 vs. Day 5), normalized to the whole brain PET images of the corresponding groups were randomly permuted to make pseudo-random groups reassigned 10,000 times and from each pseudo-group of rats, correlation matrices were calculated. The differences between the pseudorandom matrices were calculated for all 10,000 combinations. For each pair of regions, the value of p was determined by the comparison between the actual observed Z score difference (obtained with the original groups) and the distribution obtained from the permuted data. For multiple comparison correction, we applied false-discovery rate (FDR) at a threshold of FDR < 0.1.

## Results

**Figure 2:**
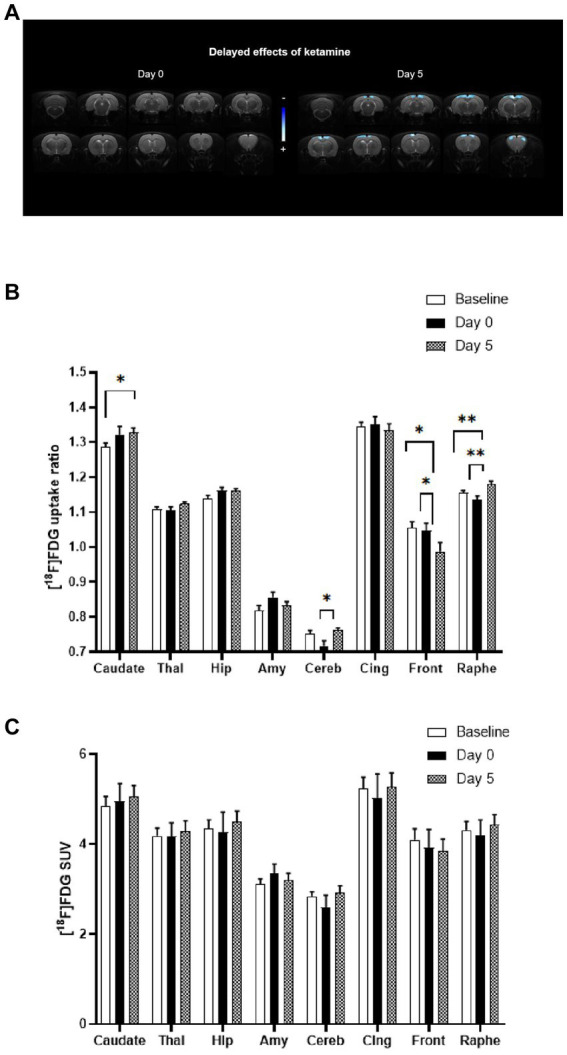
Comparison of metabolic profiles using [^18^F]FDG PET scans between acute and sustained subanaesthetic effects of ketamine (10 mg/kg, i.p.). **(A)** Voxel-to-voxel statistical comparisons of [^18^F]FDG uptake ratio between acute injection of ketamine at subanaesthetic dose (10 mg/kg, i.p.) and saline baseline in rats (*n* = 10). T scores are represented in color scales (significant decreases in blue; *p* < 0.001, Student’s *t* test). Left panel: no significant change of glucose metabolism between Day 0 (ketamine injection at subanaesthetic dose, 10 mg/kg, i.p.) and saline baseline (Day −2) in rats. Right panel: decrease of glucose metabolism between Day 5 (saline injection 5 day after ketamine injection at subanaesthetic dose) and saline baseline (Day −2) in rats (*n* = 10). Coronal sections are from +4 to −13 mm with respect to Bregma. **(B)** [^18^F]FDG uptake ratio in several ROIs after acute ketamine injection at subanaesthetic dose (Day 0) and its sustained effect at 5 days (Day 5) in rats (*n* = 10). Baseline represents saline injection 48 h before ketamine injection. Bars are the mean ± s.e.m.; *p < 0.05, **p < 0.01, Tukey’s multiple comparisons test, following two-way ANOVAs; region factor: *F*(2.367, 21.30) = 359.2, *p* < 0.0001; treatment factor: *F*(1.923, 17.31) = 0.7752, *p* = 0.4713; interaction factor: *F*(3.599, 28.28) = 5.186, *p* = 0.0037. Thal, Thalamus; Hip, Hippocampus; Amy, Amygdala; Cereb, Cerebellum; Cing, Cingulate cortex; Front, Frontal cortex. **(C)** [^18^F]FDG mean SUVs in several ROIs after acute ketamine injection at sub-anaesthetic dose (Day 0) and its sustained effect at 5 days (Day 5) in rats (*n* = 10). Baseline represents saline injection 48 h before ketamine injection. Bars are the mean ± s.e.m.; Tukey’s multiple comparisons test, following two-way ANOVAs; region factor: *F*(2.253, 20.27) = 128.8, *p* < 0.0001; treatment factor: *F*(1.143, 10.28) = 0.07003, *p* = 0.8285; interaction factor: *F*(1.569, 13.23) = 1.380, *p* = 0.2789. Thal, Thalamus; Hip, Hippocampus; Amy, Amygdala; Cereb, Cerebellum; Cing, Cingulate cortex; Front, Frontal cortex.

In Figure 2A, the statistical comparison of [^18^F]FDG uptake ratios between rats receiving acute ketamine and saline injections is displayed on the left-side using a voxel-to-voxel approach. There was no significant difference observed between baseline and bolus injection of ketamine at subanesthetic dose (Day 0). Nevertheless, alterations in the metabolic pattern appeared with a delay of 5 days following ketamine administration (on the right side). Notably, significant clusters of hypometabolism (in blue) were identified bilaterally in cortical regions, particularly in the cingulate and frontal cortex.

Similarly, results from the ROIs analysis (Figure 2B) also showed that no significant difference was found between baseline and ketamine bolus injection (Day 0). The effects in terms of metabolic pattern of the molecule at Day 5 are in line with the voxel-based analysis: a significant decrease in metabolism in the frontal cortex between Baseline and Day 5 (−6.4%, *p* < 0.05) and between Day 0 and Day 5 (−5.8%, p < 0.05) was found. A decrease in the cingulate cortex between Baseline or Day 0 and Day 5 was also observed (ns).

Significant increases in [^18^F]FDG uptake ratio were found in some ROIs, that were not apparent in the voxel-to-voxel analysis. A significant increase between Baseline and Day 5 (+2%, *p* ≤ 0.01) and between Day 0 and Day 5 (+3.8%, *p* ≤ 0.01) were found in the raphe suggesting a time-lagged effect of ketamine. In addition, a significant increase was detected in caudate (+3.3%, *p* < 0.05) between Baseline and Day 5. An increase was also found in the cerebellum between Day 0 and Day 5 (+6.4%, *p* < 0.05) and in the thalamus (ns) and the hippocampus (ns).

We also compared the raw SUVs values between the conditions and found no significant difference when no whole-brain normalization was performed, in the ROIs as well as in the whole brain (Figure 2C).

### Metabolic connectivity

**Figure 3:**
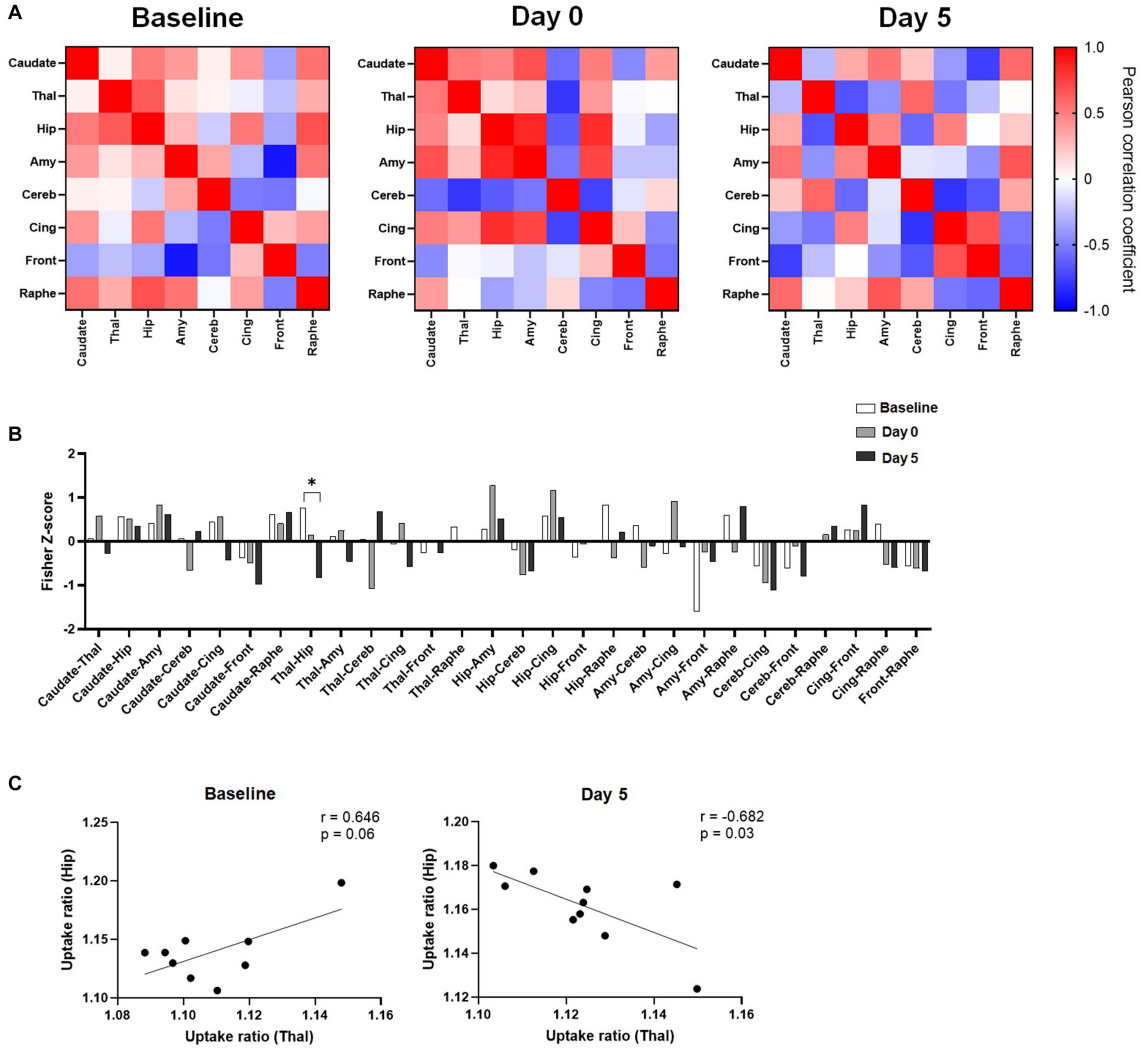
Effects of ketamine on metabolic connectivity assessed by [^18^F]FDG PET. **(A)** Pairwise connectivity matrices for each group, obtained by correlation analysis of individual uptake ratio values between the different ROIs. **(B)** Metabolic connectivity displayed as Fisher-Z scores for all pairs of regions; Significant result shown for *q < 0.1 after the permutation test and FDR correction. **(C)** Scatter plot and linear regression showing the relationship between uptake ratios in the hippocampus and in the thalamus, at baseline and 5 days after ketamine injection.

To further study the effects of ketamine, metabolic activities of regions of interest were correlated to each other, and the changes in regional pairwise correlations across rats after ketamine injection were evaluated. As shown in Figure 3A, regional correlation matrices were very different between the three time points. The permutation analysis on the Fisher Z-scores differences between groups showed a significant decrease of metabolic connectivity between the hippocampus and the thalamus at Day 5 compared to the baseline (Figure 3B). Indeed, as further illustrated in Figure 3C, the uptake ratios in these two regions at baseline tended to be positively correlated (*r* = 0.646, *p* = 0.06), whereas 5 days after ketamine administration, a negative correlation was found (*r* = −0.682, *p* = 0.03). No other significant metabolic connectivity changes were found.

## Discussion

### Acute effects of ketamine

In this study, no significant difference was observed in brain glucose metabolism following acute subanaesthetic ketamine injection in Sprague–Dawley rats and this in terms of [^18^F]FDG uptake ratio or metabolic connectivity. These findings are to be compared with previous studies that are limited and conflicting in both animals and humans. For example, Saur et al. (2017) reported no changes in rats, whereas Radford et al. (2018) found an increase in [^18^F]FDG uptake in regions such as the amygdala, hippocampus, and hypothalamus, or a decrease in the cerebellum. In humans, a cortical increase was typically observed, such in ACC, while a decrease was seen in regions like the habenula or amygdala (Carlson et al., 2013; Li et al., 2016; Chen et al., 2018; Ionescu et al., 2018). In the prefrontal cortex, studies described positive (Li et al., 2016) or negative changes (Carlson et al., 2013).

### Delayed effects of ketamine

Interestingly, the differences in metabolic profiles appeared in a delayed manner, 5 days after ketamine administration. We found a glucose uptake decrease in the frontal cortex, supporting the hypothesis that this region may be involved in the pharmacological delayed effects of ketamine. Excitatory/inhibitory (E/I) imbalance in this area has been suggested to contribute to depressive symptoms, although whether the E/I is increased or decreased following MDD, or chronic stress is still debated. Overall, many studies support the concept of a pyramidal hyperactivity in the mPFC in depressive state (Mayberg et al., 2005; Bittar and Labonté, 2021). Our findings suggest that ketamine pharmacological activity might be linked to an overall inhibition of activity in the mPFC, contributing to a normalization of the E/I balance. Interestingly, synaptic plasticity has been suggested to be involved in the antidepressant long-lasting effects of ketamine (Li et al., 2010). This hypothesis has been recently investigated in human using PET imaging of synaptic density (Holmes et al., 2022). Overall, no significant changes of SV2A density were observed 24 h after administration in healthy controls and patients, but some individuals that displayed initial low SV2A density displayed increased uptake after ketamine. Although in a different species, our findings raise the possibility that the sustained effects of ketamine might be more obvious only several days after administration. Nevertheless, it would be valuable to evaluate the correlation between glucose uptake changes and synaptic density in the same individuals after ketamine administration in future PET studies, to understand the relationship between synaptic density and cerebral glucose uptake in patients and animal models. In addition, future studies could use specific antagonists to evaluate the different molecular contributions to the effect of ketamine of synaptic plasticity, as the TrkB antagonist ANA-12 for the BDNF–TrkB pathway.

Interestingly, an increase in glucose uptake was found in the dorsal raphe nuclei (DRN) region in this study, suggesting a contribution of the serotonergic system in the delayed action of ketamine, and in line with some previous findings (Gigliucci et al., 2013; Fukumoto et al., 2018; Pham and Gardier, 2019). In addition, and in accordance with clinical data, we found an increase in glucose uptake at the striatum level. Studies have shown that ketamine administration can lead to an increase in dopamine release in the striatum (Kokkinou et al., 2018; Sterpenich et al., 2019). This might also support its therapeutic effect since dysregulation of the dopamine system in the striatum may play a role in the development of as depression (Grace, 2016).

Our results did not find any significant effect of ketamine in glucose uptake in the limbic system (such as the cingulate cortex or hippocampus), which could also be involved in the antidepressant action of ketamine (Vollenweider and Kometer, 2010; Aleksandrova et al., 2017). However, we measured a significant reduction in metabolic connectivity between the hippocampus and the thalamus, 5 days after ketamine administration, although the glucose uptake ratio tended to increase in both regions. The functional relevance of this observation is unclear but consistent with the numerous findings in the literature showing that ketamine modulates the connectivity between the thalamus and other regions (Dawson et al., 2013; Höflich et al., 2015; Liao et al., 2016; B Hughes et al., 2020). The complex interaction between thalamus and hippocampus under ketamine influence was previously studied using local field potentials recordings in rats, showing that ketamine effects in the hippocampus are mediated through both thalamus-dependent and independent mechanisms, preferentially the latter at lower dose (Zhang et al., 2012). Still, this does not fully explain the delayed negative correlation that we observed, which deserves further investigation.

### Study limitations

There are several limitations for the present study that should be acknowledged. Healthy rats were used, and the next study should be performed on animal models presenting a ‘depression-like’ phenotype, such as the chronic mild stress or chronic corticosterone models. In this study, the administered molecule was a ketamine racemic. Studies has shown that the actions of R-ketamine and S-ketamine are distinct (Masaki et al., 2019; McMillan and Muthukumaraswamy, 2020; Bonaventura et al., 2021). Additionally, it is essential to highlight the differences in the administration methods used across studies. In this case, intraperitoneal administration was chosen for practical reasons, but the pharmacokinetics and pharmacodynamics may vary when using other routes of administration, such as nasal or infusion. Moreover, future studies should be conducted with repeated administrations of ketamine or additional time-points (later and earlier) to elucidate the onset and duration of the effects. Static data acquisition was chosen (no kinetic modeling was performed) to enable awake [^18^F]FDG uptake. Indeed, uptake of this radiotracer in anaesthetized animals can strongly alter measurements (Levigoureux et al., 2019). For improved robustness of the findings, changes occurring in 5 days after injection of saline could have been studied, but we chose to focus on the longitudinal comparisons in a single group of animals in this proof-of-concept study, in order to reduce the number of animals (each subject serves as its own control). For questions of reproducibility, an injection was carried out at Baseline, Day 0 and Day 5, since stress can induce variations in glucose metabolism and therefore in [^18^F]FDG uptake.

## Conclusion

In summary, this research extends the existing literature on ketamine mechanism of action as a rapid-acting antidepressant, which has currently little explored the late effects of a single dose of ketamine. By its longitudinal aspect, the present study was able to demonstrate the sustained and prolonged effects of ketamine on cerebral glucose metabolism. The frontal cortex, cerebellum, striatum, and DRN were found to be the main regions affected by ketamine’s prolonged activity. Furthermore, the connectivity analysis revealed a shift in the limbic system, characterized by a decrease in metabolic connectivity between the thalamus and hippocampus. This study suggests that [^18^F]FDG-PET is useful to evaluate the sustained effects of ketamine, which could be further used to this aim in human studies, given the easy accessibility to this radiotracer.

## Data availability statement

The raw data supporting the conclusions of this article will be made available by the authors, without undue reservation.

## Ethics statement

The animal study was reviewed and approved by Comité d éthique pour l expérimentation animale neurosciences Lyon (CELYNE).

## Author contributions

SC carried out the experiments with PET imaging, performed data analysis, participated in the study design, and wrote the manuscript. CB carried out the experiments with PET imaging. SB participated in its design and reviewed the manuscript. BV performed metabolic connectivity data analysis and reviewed the manuscript. LZ initiated the study, participated in its design, and reviewed the manuscript. EL coordinated the study, carried out the experiments, participated in its design, and reviewed the manuscript. All authors have read and approved the final manuscript.

## Funding

This work was supported by the LABEX PRIMES (ANR-11-LABX-0063) of Université de Lyon and the program “Investissements d’Avenir” (ANR-11-IDEX-0007) operated by the French National Research Agency (ANR) and by the imaging platform, CERMEP (Lyon, France).

## Conflict of interest

The authors declare that the research was conducted in the absence of any commercial or financial relationships that could be construed as a potential conflict of interest.

## Publisher’s note

All claims expressed in this article are solely those of the authors and do not necessarily represent those of their affiliated organizations, or those of the publisher, the editors and the reviewers. Any product that may be evaluated in this article, or claim that may be made by its manufacturer, is not guaranteed or endorsed by the publisher.
